# New Approaches to Studying Silent Mesial Temporal Lobe Seizures in Alzheimer's Disease

**DOI:** 10.3389/fneur.2019.00959

**Published:** 2019-09-04

**Authors:** Alice D. Lam, Andrew J. Cole, Sydney S. Cash

**Affiliations:** ^1^Massachusetts General Hospital, Department of Neurology, Boston, MA, United States; ^2^Harvard Medical School, Boston, MA, United States

**Keywords:** foramen ovale electrode, Alzheimer, epilepsy, temporal lobe, machine learning

## Abstract

Silent seizures were discovered in mouse models of Alzheimer's disease over 10 years ago, yet it remains unclear whether these seizures are a salient feature of Alzheimer's disease in humans. Seizures that arise early in the course of Alzheimer's disease most likely originate from the mesial temporal lobe, one of the first structures affected by Alzheimer's disease pathology and one of the most epileptogenic regions of the brain. Several factors greatly limit our ability to identify mesial temporal lobe seizures in patients with Alzheimer's disease, however. First, mesial temporal lobe seizures can be difficult to recognize clinically, as their accompanying symptoms are often subtle or even non-existent. Second, electrical activity arising from the mesial temporal lobe is largely invisible on the scalp electroencephalogram (EEG), the mainstay of diagnosis for epilepsy in this population. In this review, we will describe two new approaches being used to study silent mesial temporal lobe seizures in Alzheimer's disease. We will first describe the methodology and application of foramen ovale electrodes, which captured the first recordings of silent mesial temporal lobe seizures in humans with Alzheimer's disease. We will then describe machine learning approaches being developed to non-invasively identify silent mesial temporal lobe seizures on scalp EEG. Both of these tools have the potential to elucidate the role of silent seizures in humans with Alzheimer's disease, which could have important implications for early diagnosis, prognostication, and development of targeted therapies for this population.

## Introduction

The recognition of subclinical seizures and spiking in a genetic mouse model of Alzheimer's disease (AD) over 10 years ago was a compelling finding, which demonstrated that network hyperexcitability and epileptiform activity can arise spontaneously in AD without any obvious clinical manifestations (1). Subsequent work has advanced the therapeutic hypothesis that aberrant electrical activity could be a modifiable contributor to disease pathology and cognitive impairment in AD (2–5).

Historically, the role of aberrant electrical activity in human AD has received relatively little attention. While it is clear that people with AD have an increased risk of developing seizures and epilepsy (6–9), the current clinical approach to treating seizures in AD is based on the assumption that seizures are a late side effect of neurodegeneration that should only be treated when they arise clinically. Yet, epidemiologic studies have demonstrated that clinical seizures can manifest early in the course of AD, often preceding the onset of cognitive decline (10, 11). Moreover, studies using prolonged scalp EEG recordings have demonstrated that subclinical epileptiform abnormalities can occur in 20–40% of AD patients with no known history of clinical seizures (12, 13) and may be associated with a faster rate of cognitive decline (12). Growing evidence from animal models of AD has now established a feed-forward mechanism linking neuronal hyperactivity with the deposition and propagation of amyloid plaques and neurofibrillary tangles (14–21). Therefore, an important question is whether there are significant changes in brain electrical activity that arise early in the disease course, without clinical symptoms, that nonetheless play a role in memory impairment or clinical progression of AD.

Here, we define “silent” epileptiform abnormalities in humans as epileptiform abnormalities (seizures or spikes) that are both: (1) *subclinical* (occurring without any obvious clinical signs or symptoms) and (2) *not visibly epileptiform on scalp EEG*. We will focus here on silent epileptiform abnormalities that arise specifically from the mesial temporal lobe (mTL, e.g., amygdala, hippocampus, entorhinal cortex), the earliest brain structure involved in AD and one of the most epileptogenic regions of the brain – in short, the brain structure from which epileptiform abnormalities are most likely to arise early in the course of AD.

The concept of silent epileptiform abnormalities is well-known in the field of epilepsy, where intracranial electrodes have been used for decades to study patients with medically intractable epilepsy (22, 23). Intracranial electrode recordings can shed light on epileptiform abnormalities from deep brain structures that would have never been suspected based on scalp EEG recordings alone. Because the mTL plays a central role in many forms of epilepsy, it is one of the brain structures that has been studied most extensively with intracranial electrode recordings. Studies that evaluate the scalp EEG correlates of mTL seizures and spikes identified on intracranial electrode recordings demonstrate that the majority of mTL epileptiform activity occurs without any clear semblance of epileptiform activity on scalp EEG (24–32).

We hypothesize that silent mTL epileptiform abnormalities play an important role in impairing cognition in early stages of AD. While this hypothesis lies outside the current molecular framework for understanding AD, it nevertheless could address several clinical features of AD that are not currently accounted for by the amyloid hypothesis. First, it could account for the fluctuating course of “good days and bad days” often described by patients and their families early in the clinical phase of the disease. Second, it could account for the anatomic specificity of the mesial temporal lobe (mTL) as the starting point of the disease. The clinical and electrographic challenges of identifying silent mTL epileptiform activity have unfortunately made it difficult to test this hypothesis in AD patients.

In this perspective, we will describe two new approaches for studying silent mTL epileptiform activity in AD, both of which draw heavily on our experience in patients with epilepsy. The first approach, *intracranial recordings with foramen ovale electrodes*, is a minimally invasive technique that was developed to record mTL epileptiform activity in patients with suspected temporal lobe epilepsy. The second approach, using *machine learning to non-invasively detect mTL epileptiform activity from scalp EEG recordings*, is informed by large datasets of combined intracranial and scalp EEG recordings from patients with temporal lobe epilepsy. Combining both approaches has the potential to shed light on the role of silent mTL epileptiform abnormalities in human AD, opening the door to novel diagnostic, prognostic, and therapeutic avenues for this disease.

## Intracranial Recordings With Foramen Ovale (FO) Electrodes

### Placement of FO Electrodes for Recording mTL Activity

Placement of bilateral intracranial foramen ovale (FO) electrodes is a clinical procedure that has been used worldwide in the pre-surgical evaluation of both children and adults with suspected mTL epilepsy (28, 33–37). The technique was first described by Wieser et al. (37), as a method to directly assess electrical activity from the mesio-basal temporal lobe. Each mTL is recorded by a single multi-contact FO electrode, which is inserted through the ipsilateral cheek and guided into the cranium through the ipsilateral foramen ovale (a naturally occurring opening at the base of the skull), using fluoroscopy. The FO electrodes are positioned to lie in the ambient cistern (the CSF space directly adjacent to the mTL), where they record mTL activity with high fidelity. Placement of bilateral FO electrodes is typically performed in the operating room with the patient under general anesthesia. The entire procedure typically takes <1.5 h.

**Figure 1:**
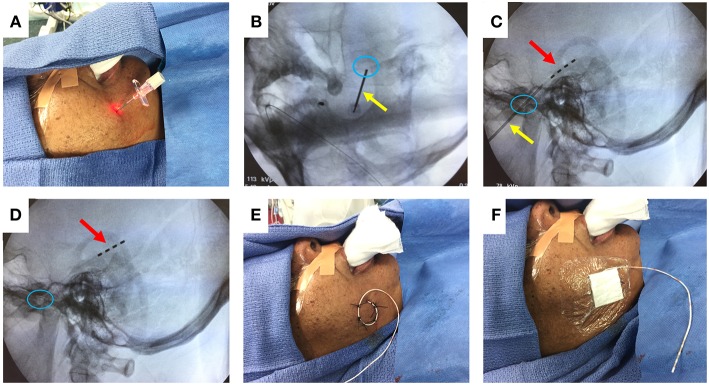
FO electrode placement procedure. **(A)** Introducer needle inserted through cheek skin. **(B)** Fluoroscopy image (oblique view) showing needle (yellow arrow) passing through the foramen ovale (blue circle). **(C)** Fluoroscopy image (lateral view) showing advancement of 4-contact FO electrode (red arrow) past the needle tip. **(D)** Fluoroscopy image (lateral view) showing removal of the needle, leaving the FO electrode in place. **(E)** Sutures securing the FO electrode to the cheek. **(F)** Sterile dressing placed over the FO electrode. Written informed consent for the use of these images was obtained from the individual shown in the images.

Figure 1 illustrates the steps to this procedure, which has previously been described in detail (33, 37). The cheek is first prepped with chlorhexidine solution and injected with 1% lidocaine for local anesthesia. Under fluoroscopy, the ipsilateral foramen ovale is identified. A 16-gauge introducer needle is used to pierce the cheek skin approximately 2.5 cm lateral to the labial fissure. Guided by fluoroscopy, a 10-cm needle with a stylet is advanced through the skin and soft tissue, toward and then through the foramen ovale. The final targeted position of the needle tip is approximately 2 mm anterior to the clival line. The stylet is withdrawn, and a 4-contact, 1 mm diameter FO electrode (Ad-Tech, Racine WI) is advanced through the needle, so that all electrode contacts are positioned just beyond the tip of the needle. A lateral fluoroscopic image is obtained to evaluate the final position of the electrode, and the needle is withdrawn. The FO electrode is secured to the cheek using 0 silk sutures, and a sterile dressing is placed over the electrode exit site. The procedure is then repeated on the contralateral side.

**Figure 2:**
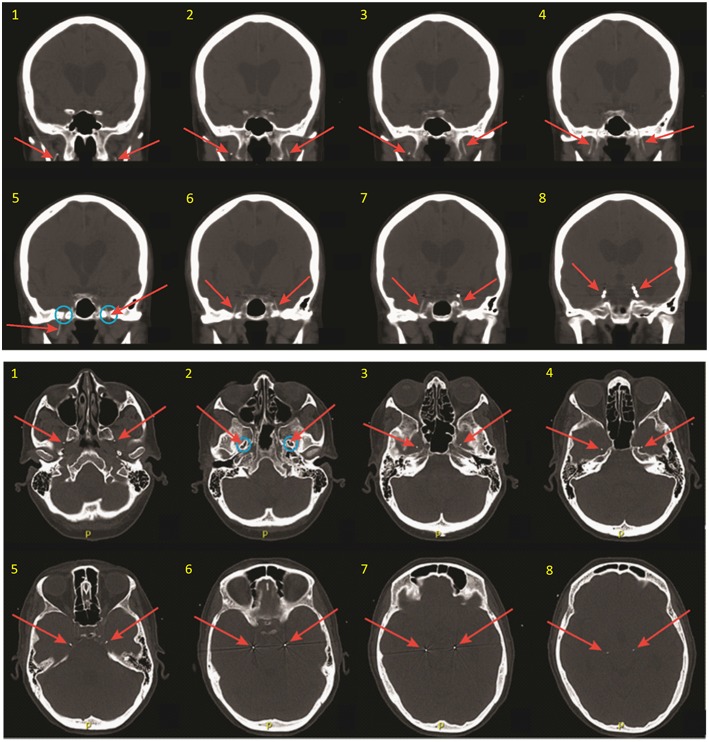
Coronal (**top**) and thin axial (**bottom**) views on post-operative CT scan, demonstrating FO electrode positioning. Slices are numbered in ascending order from anterior to posterior (coronal images), and from inferior to superior (axial images). Red arrows show the FO electrodes in each image. Note that the FO electrodes are only 1 mm in diameter but appear larger in these images due to image slice thickness and windowing needed for visualization. Blue circles show the foramen ovale.

After placement of both FO electrodes, patients are woken from general anesthesia and monitored closely in the post-anesthesia recovery room for ~1 h. A non-contrast head CT is obtained to verify the final position of the electrodes relative to the mTL (Figure 2), and the patient is then transferred to the inpatient Epilepsy Monitoring Unit for further monitoring while the FO electrodes are in place. Patients are typically given antibiotic prophylaxis (vancomycin and ceftriaxone) peri-operatively and over the first 48 h of hospitalization to prevent infection. A benefit of FO electrode recordings over other types of intracranial electrodes is that a full set of standard scalp EEG electrodes can be placed and simultaneously recorded from, without concern for skull defects (e.g., burr holes or craniotomies) affecting placement of the scalp electrodes or causing distortion of scalp EEG signals due to breach effect.

Patients can remain implanted with FO electrodes for weeks at a time, though implantation times >8 days are associated with increased morbidity (35). Once all the necessary information has been obtained from the FO electrode recordings, removal of FO electrodes is performed at the patient's bedside. This is a painless procedure that does not require sedation or local anesthesia. The silk sutures are cut, and the electrodes are gently withdrawn by hand. Patients are monitored closely for a few hours afterwards for development of headache or other neurological deficits. If no symptoms develop, patients can be discharged home on the same day.

### Comparison of FO Electrodes With Other Recording Techniques

Compared to other intracranial recording methods such as stereotactic depth electrodes or subdural grids and strips, placement of FO electrodes is considered minimally invasive. Unlike these other methods, placement of FO electrodes does not require a craniotomy or drilling burr holes into the skull; the skull remains entirely intact. Moreover, FO electrodes do not need to penetrate brain tissue or make direct contact with the brain in order to record mTL activity. Rather, they float in the CSF space adjacent to the mTL.

FO electrodes are commonly confused with extracranial recording methods such as nasopharyngeal or sphenoidal electrodes. While nasopharyngeal and sphenoidal electrodes can be placed at the bedside, these are not intracranial electrodes, and they lack the ability to reliably capture electrical activity from the mTL (28, 38). On the other hand, 70–95% of the epileptiform discharges identified on FO electrodes cannot be seen on scalp EEG, and entire mTL seizures can be recorded on FO electrodes, without a visible epileptiform change on scalp EEG (24, 27, 28, 39, 40) Magneto-encephalography (MEG) has also been used as a non-invasive tool to assess mTL activity in epilepsy with varying results (29, 41–43). From a practical standpoint, however, MEG cannot be used to record for more than a few hours at a time, and performing overnight MEG recordings to capture natural sleep or rare paroxysmal events would be nearly impossible.

A major limitation of FO electrodes is that they cannot be used to assess electrical activity from brain regions other than the mTL. For this reason, recording with FO electrodes has largely fallen out of favor in the workup of medication-refractory epilepsy, where most epileptologists prefer to use more invasive intracranial recording techniques that permit a wider sampling of brain regions, in order to definitively localize the seizure onset zone. However, for patients in whom there is a high suspicion for mTL epilepsy, but where there are specific questions related to the laterality of mTL seizures, FO electrodes can offer a simple and direct test of these hypotheses (33, 36).

For the purposes of studying silent mTL epileptiform activity in patients with AD, we believe that recording with FO electrodes is the current gold standard. No other available and less invasive recording technique offers the ability to obtain high quality, long-term recordings directly from the mTL. Moreover, as the mTL is the primary site of interest in this case, the limitation that FO electrodes are only able to sample from the mTL becomes irrelevant.

### FO Electrode Recordings in Alzheimer's Disease

In 2017, our group reported the first intracranial electrode recordings in AD patients, using bilateral FO electrodes (39). We studied two patients, one with amnestic mild cognitive impairment (aMCI), and the other with moderate dementia, both of whom presented with fluctuations in cognition or behavior. Both patients had cerebrospinal fluid biomarker-confirmed AD. As part of their clinical workup, they underwent recordings with combined scalp EEG and FO electrodes.

**Figure 3:**
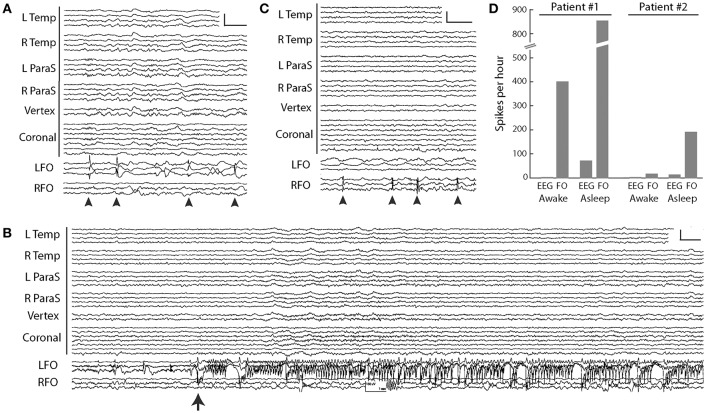
FO electrode recordings in two AD patients. **(A)** Silent mTL spikes (arrowheads) seen on the left FO electrode in the MCI patient. Scale: 200 μV, 1 s. **(B)** Silent mTL seizure arising seen on the left FO electrode in the MCI patient. Scale: 150 μV, 1 s. **(C)** Silent mTL spikes (arrowheads) seen on the right FO electrode in the patient with moderate dementia. Scale: 200 μV, 1 s. **(D)** Spike frequencies observed on scalp EEG and FO electrodes during wakefulness and sleep, in the two AD patients. Patient #1 had aMCI and Patient #2 had moderate dementia. In **(A–C)**, scalp EEG is shown as a longitudinal anterior-posterior bipolar montage. L Temp, left temporal (Fp1–F7, F7–T3, T3–T5, T5–O1); R Temp, right temporal (Fp2–F8, F8–T4, T4–T6, T6–O2); L ParaS, left parasagittal (Fp1–F3, F3–C3, C3–P3, P3–O1); R ParaS, right parasagittal (Fp2–F4, F4–C4, C4–P4, P4–O2); Vertex (Fz–Cz, Cz–Pz); Coronal, coronal ring (T1–T3, T3–C3, C3–Cz, Cz–C4, C4–T4, T4–T2, T2–T1); LFO, left FO electrode (LFO1–LFO2, LFO2–LFO3, LFO3–LFO4); RFO, right FO electrode (RFO1–RFO2, RFO2–RFO3, RFO3–RFO4). FO1 is the deepest contact. Figure adapted with permission from Lam et al. (39). Written informed consent for the publication of these cases was not required as this information is publicly available (39).

The aMCI patient was a 67-year-old woman who had noted worsening memory over the prior year, including unusual spells of confusion lasting days at a time. Her FO electrode recordings demonstrated abundant, sleep-activated spikes (Figure 3A). Eighty-five percent of these spikes arose from the left mTL, while 15% arose from the right mTL. Over 95% of the spikes seen on FO electrodes could not be seen on the corresponding scalp EEG. mTL spikes were most prevalent during non-REM sleep, occurring at an average rate of ~900 spikes per hour during N3, ~740 per hour during N2, and ~670 per hour during N1. In comparison, average mTL spike rates were only ~330 per hour during wakefulness and ~160 per hour during REM sleep (44). During the first 12 h of recording, the FO electrodes captured 3 silent mTL seizures, all occurring during sleep, and all occurring without any visible evidence of seizures on scalp EEG (Figure 3B). The patient and her husband had no awareness of these events. The patient was started on levetiracetam the following day, resulting in a 65% reduction in overall mTL spike rate and no further seizures. After treatment with levetiracetam 750 mg twice daily, her confusional episodes largely resolved, with the exception of two occasions where she forgot to take her medication. Three years later, she has progressed to a stage of mild dementia.

Our second patient was a 63-year-old woman with moderate dementia, who had developed memory problems at age 58. She was noted to have a fairly rapid cognitive decline over the prior year and presented at age 63 with new-onset fluctuations in her anxiety levels throughout the day. Her FO electrode recordings demonstrated frequent sleep-activated spikes arising exclusively from the right mTL, with average rates of 30–80 spikes per hour during non-REM sleep, compared to rates of 11 per hour during wakefulness (REM sleep was not captured) (44) (Figure 3C). Ninety-five percent of the spikes seen on FO electrodes were not visible on scalp EEG. During her hospitalization, she required frequent redirection and reminders not to pull on her EEG or EKG wires; despite this, she partially removed the right FO electrode within 24 h of FO electrode implantation. Given concern that she would also dislodge the left FO electrode, this was removed by medical staff on the following day. The patient was started on levetiracetam 500 mg twice daily on discharge from the hospital but did not tolerate this due to behavioral side effects. She continued to decline cognitively and passed away 2 years later following a sudden cardiac arrest.

A comparison between the FO electrode recordings of our patients raises a few interesting observations and hypotheses, though at the potential risk of over-interpreting our very limited data. First, there was a striking difference in mTL spike rates between our two patients, with the aMCI patient exhibiting an up to 10-fold increased spike rate compared to the patient with moderate dementia (Figure 3D). Moreover, our aMCI patient had several silent mTL seizures, whereas our patient with moderate dementia did not. These findings could be congruent with the concept that the earliest stages of AD are associated with a greater extent of neuronal hyperactivity compared to later stages of disease, which has been born out in both fMRI studies of hippocampal activity during memory tasks in humans, as well as in *in vitro* and *in vivo* studies of neuronal activity in mouse models of AD (45). Second, we found that mTL spikes from the aMCI patient were largely lateralized to the left mTL, whereas in the moderate AD patient, they lateralized to the right mTL. Whether there is an early predilection of left hemispheric hyperactivity in AD is unclear. Long-term scalp EEG results from AD patients reported by Vossel et al. (12), as well as our own ambulatory scalp EEG data in patients with AD (manuscript under review), also corroborate a left hemispheric asymmetry in subclinical epileptiform activity in these patients, though spikes seen on scalp EEG may not reflect the same process as spikes seen on FO electrode recordings. Interestingly, there is imaging data from both anatomical and functional connectivity modalities to support a susceptibility of the left mTL in early stages of AD, that may be mediated in part by the APO-E4 allele (46–53). Whether these findings are simply related to ascertainment bias (i.e., AD patients with dominant hemisphere pathology may be more likely to present with memory deficits) is unclear.

### Special Considerations Regarding FO Electrode Recordings in Alzheimer's Disease

Placement of FO electrodes in AD patients warrants special considerations, particularly given the older age, medical comorbidities, and cognitive impairments that are typical of this population. These concerns are outlined below.

1. *Higher risk of peri-operative delirium and delirium associated with the inpatient hospital stay*. Selecting patients with less advanced stages of AD (mild cognitive impairment or mild dementia) and with minimal baseline agitation may reduce this risk. Precautions to reduce risk of delirium in this population should be taken (54, 55). Medications that can worsen delirium, including anti-cholinergics, benzodiazepines, and narcotics, should be avoided. Unnecessary labs and monitoring of vital signs overnight should also be minimized to prevent sleep disruption. Family members and nursing staff should be encouraged to be present to re-assure and re-orient the patient as needed during the hospital stay. Placement of familiar objects or pictures around the hospital room may also be helpful.
2. *Higher risk of patients pre-maturely removing the FO electrodes due to cognitive impairment*. As above, this can be minimized by selecting patients with less advanced stages of AD. Frequent supervision and re-direction, either by nursing staff and/or family members, particularly during overnight hours when sundowning may occur, may also reduce this risk.
3. *Medical co-morbidities*. AD patients are more likely to be medically frail compared to a younger epilepsy patient. Cardiac or pulmonary disease may also be more prevalent given the older age of AD patients and may preclude these patients from safely undergoing general anesthesia for the procedure. All patients should be evaluated pre-operatively to ensure that it is medically safe for them to undergo general anesthesia. Concurrent treatment with anticoagulant medications may also place patients at greater risk for serious adverse events from FO electrode placement. Patients should be assessed on an individual basis to determine whether it is safe to hold the anticoagulant medication peri-operatively and while FO electrodes are in place. If this is not deemed to be safe, we would discourage implanting these patients with FO electrodes, particularly for a research study.
4. *Concern that general anesthesia may precipitate a worsening in cognition or accelerate disease progression in AD patients*. A body of literature from animal models of AD suggests a potential relationship between anesthesia and neurotoxicity, though definitive evidence of general anesthesia hastening clinical progression of AD in human is lacking (55, 56). Moreover, there is a paucity of data on how best to minimize this potential risk. The brief length of the FO electrode placement procedure (typically <1.5 h) may reduce this risk compare to longer surgical procedures.
5. *Ethical considerations regarding capacity to consent to the procedure*. The ethical issues surrounding consent for FO electrode placement in AD patients bear some similarity to those that have previously been addressed in clinical trials studying deep brain stimulation in AD patients (57). Continued re-evaluation of the capacity for decision-making in patients with AD is critical, as this ability may vary from day to day, and will decline over time (58). Given the complexity and potential risk of FO electrode studies, we recommend that a cognitively normal study partner (typically the patient's health care proxy) be present for all study procedures and provide informed consent for the procedure if the person with AD lacks capacity to do so.
6. *Definitive diagnosis vs. empiric treatment?* One could argue whether placement of FO electrodes to definitively diagnose silent mTL epileptiform activity in people with AD is really necessary, or whether people with AD could just be treated empirically with anti-seizure medications. While easy to implement, empiric treatments are fraught with problems that ultimately limit their utility in the long run. An empiric approach provides essentially no information on (a) how common silent mTL epileptiform abnormalities are in AD; (b) how to identify the subset of AD patients who may have this abnormality; and (c) whether medications being offered are appropriate and effective for treating silent mTL epileptiform activity. For example, it is possible that a small but significant subset of AD patients (e.g., 10–15%) have silent mTL seizures and that this small subset would benefit greatly from early treatment with seizure medications. The signal of this benefit is likely to be lost in a study that empirically administers treatment to a much larger population of AD patients, the majority of whom would not have any benefit. Thus, rather than empirically treat the entire population, it is essential to first define the sub-population that stands to benefit from this treatment. It remains unclear as to whether treatment of silent mTL seizures will result in improvement in cognition or reduce the rate of clinical progression in AD. Evaluating this hypothesis requires first detecting and then suppressing these seizures.

## Artificial Intelligence Approaches to Studying Mesial Temporal Epileptiform Activity

Further studies with FO electrode recordings in AD patients are clearly needed, to better understand the role of silent mTL epileptiform activity in AD. However, as a clinical diagnostic tool for AD patients in the long run, performing FO electrode recordings on a large scale is not feasible for a number of reasons, including cost, requirement for specialized expertise and hospital resources to perform the monitoring, and willingness of patients to undergo a surgical procedure for diagnostic purposes. As such, there is a need to develop non-invasive and inexpensive methods for diagnosis of silent mTL epileptiform activity that can be widely implemented.

While a number of approaches based on different neurophysiologic or imaging modalities are possible, here we will focus on describing a computational approach that we have been developing to non-invasively detect silent mTL epileptiform activity from scalp EEG recordings. The basic premise of this work is that while silent mTL epileptiform activity lacks an obvious (visible) epileptiform correlate on scalp EEG, there may be associated subtle, quantitative changes reflected on the scalp EEG that would allow this activity to be detected indirectly. Many studies have now demonstrated that mTL spikes and seizures induce changes in both local and long-distance networks (59–64), and we hypothesize that these changes in network activity could be used to define a scalp EEG-based “signature” of silent mTL epileptiform activity. We use artificial intelligence methods to “learn” these scalp EEG signatures of mTL epileptiform activity.

**Figure 4:**
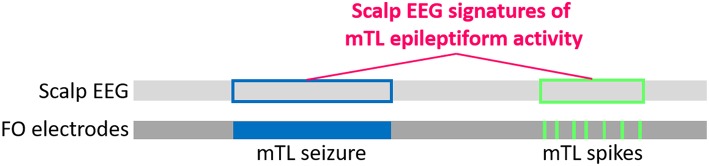
Schematic for developing scalp EEG-based detectors of silent mTL epileptiform activity, using datasets of simultaneous scalp EEG and FO electrode recordings from TLE patients. The light and dark gray bars represent a simultaneously captured scalp EEG and FO electrode recording, with the horizontal direction representing time. Silent mTL seizures (filled blue box) and spikes (filled green boxes) can be identified on visual analysis of the FO electrode recordings, and the times at which these events occur can be easily determined. We translate this timing information to the corresponding scalp EEG (unfilled blue and green boxes, respectively) and apply signal processing and machine learning approaches to learn the scalp EEG signatures of the underlying silent mTL epileptiform activity.

Our approach uses a large clinical dataset of simultaneous scalp EEG and FO electrode recordings from patients with temporal lobe epilepsy (TLE) who were previously evaluated at our institution. The FO electrodes provide the gold standard to determine when silent mTL epileptiform activity occurred, and we use the corresponding scalp EEG signal to identify signatures of this silent mTL epileptiform activity (Figure 4). The rationale behind using data from TLE patients is that these patients, like AD patients, also demonstrate silent mTL epileptiform activity, including spikes and seizures that are visible on FO electrodes but not on the scalp EEG. FO electrode recordings are frequently used in the clinical care of TLE patients with medication-refractory epilepsy as part of their evaluation for epilepsy surgery. As such, a large amount of FO electrode data from TLE patients is available and can be leveraged to provide the many examples of silent mTL epileptiform activity needed to learn the subtle scalp EEG correlates of this activity.

Notably, using FO electrode recordings for this application has an important advantage compared to datasets from other types of intracranial recordings (e.g., stereotactic depth electrodes or subdural grids/strips). Because placement of FO electrodes does not result in a skull breach (i.e., due to burr holes or craniotomy), the scalp EEG signals being analyzed here are not distorted by breach effects, as they would be with other types of intracranial electrode recordings. Thus, the scalp EEG signatures and algorithms to detect silent mTL epileptiform activity that are developed using this dataset can be applied to patients with intact skulls (i.e., most patients).

### Scalp EEG-based Detection of Silent mTL Seizures

We have recently demonstrated the feasibility of this approach to detect and lateralize silent mTL seizures with high specificity in patients with TLE (65, 66). In a proof-of-principle study, we assessed whether silent mTL seizures could be detected on scalp EEG, based on changes in network activity (65). We identified 25 silent mTL seizures in 10 patients with TLE and obtained control records from another 13 patients. We divided each patient's scalp EEG recording into 2-second epochs and extracted coherence features from each epoch. We used these coherence features as inputs to a logistic regression classifier and trained it to determine whether a 2-second EEG epoch occurred during a silent mTL seizure or not. A clustering rule was then applied to group neighboring epochs classified as seizures into a single detected event. Using a leave-one-patient-out cross-validation (which estimates how well the detector would perform on data from a new patient), the detector correctly identified and lateralized silent mTL seizures in 40% of patients with silent mTL seizures. On average, the detector identified 30% of the silent mTL seizures per patient. The detector had a positive predictive value of 75% and false alarm rate of 0.3 per day. Notably, while identifying only 40% of patients with silent mTL seizures is far from ideal, it is important to note that *none* of these patients would have been identified as having silent mTL seizures, based on our current clinical interpretation of their scalp EEG data.

An important question is whether scalp EEG-based detectors that are developed in patients with TLE can be applied meaningfully to patients with AD. Notably, one of the patients whose data was used to develop the silent mTL seizure detector [Patient #9 from (65)] was the aMCI patient described above (section FO Electrode Recordings in Alzheimer's Disease), who had 3 silent mTL seizures in the first night of FO electrode recording. On cross-validation, the silent mTL seizure detector successfully identified all 3 of her seizures, while raising only one false positive detection. While this supports the idea that detectors of silent mTL epileptiform activity developed in TLE patients can be successfully applied to AD patients, further validation of this approach in AD patients will certainly be needed.

In another publication (66), we used the same clinical dataset of simultaneous FO electrode and scalp EEG recordings from TLE patients to analyze seizures that start focally in the mTL as a silent seizure, but that then spread spatially to become visible on the scalp EEG. We asked whether the initial silent portion of the seizure could be detected and lateralized on scalp EEG. This scenario differs from the detector described above, in that it is clear from the scalp EEG that a seizure has occurred, though it is not clear whether there was initial silent mTL seizure activity preceding this, for how long, and on which side. We used a data set of 86 seizures from 29 TLE patients, with the silent mTL portion of the seizure ranging from 0 to 42 s. Seizures that had a silent mTL onset lasting at least 5 s were defined as having an occult mTL onset, whereas those with a silent mTL onset lasting <5 s were defined as not having an occult mTL onset. We extracted scalp EEG spectral power as an input feature to train machine learning algorithms to detect the silent mTL portion of these seizures. Using this algorithm, we correctly classified 73% of seizures with an occult mTL onset as having one, and we also correctly classified 100% of the seizures that lacked an occult mTL onset as lacking one. We were able to predict the length of the silent mTL seizure onset with high accuracy, with the algorithm predictions exhibiting strong correlation with the actual length of the silent mTL seizure onset (*r* = 0.69). Moreover, the algorithm allowed us to make predictions on the laterality of seizure onset for 50% of the seizures with a silent mTL onset; these predictions had an accuracy of 94%.

Altogether, the studies described above provide the proof of principle that scalp EEG data can provide important information to both identify and lateralize silent mTL seizure activity, in the absence of visible epileptiform activity, and with relatively high specificity. False positive detections, while relatively low, remain the major limitation for practical application of these algorithms for clinical or research use. Larger datasets with many more examples of silent mTL seizures are likely to result in substantial improvements in algorithm performance, however. Particularly for application in AD patients, these computational methods will require additional FO electrode recordings from AD patients, which will be critical for refining and validating these detectors (developed using data from TLE patients) for use specifically in AD.

### Scalp EEG-based Detection of Silent mTL Spikes

In addition to silent mTL seizures, the FO electrode recordings in our two AD patients also demonstrated frequent mTL spikes. A similar computational approach could also be used to develop algorithms to detect silent mTL spikes. One advantage of this is that spikes occur with much higher frequency than seizures. Spike rates are often measured on the order of minutes to hours, whereas seizure frequency is typically measured on the order of days to months. From a diagnostic standpoint, detection of silent mTL spikes may be a more efficient way to identify AD patients with mTL hyperexcitability, as it would require a shorter recording time compared to detection of silent mTL seizures. From a computational standpoint, development of algorithms to detect silent mTL spikes from scalp EEG would also benefit from a much larger dataset of spike examples on which to train, compared to seizures. On the other hand, spikes are very brief events (<70 ms in duration) compared to seizures (typically ~ 10–120 s), and whether mTL spikes generate a scalp EEG signature with a signal-to-noise ratio sufficient to allow detection of single events remains unclear.

Notably, one group, using a small dataset of combined FO electrode and scalp EEG recordings (each 20 min long) from 18 TLE patients, was able to develop an algorithm that could detect silent mTL spikes with accuracies above chance level, with an area under the curve of a receiver operating characteristic curve of 0.67 (67). While false positive detections remain the limiting factor for practical application of this algorithm, their work supports the concept that scalp EEG signals can contain important information regarding mTL epileptiform activity, even in the absence of a visible epileptiform discharge.

## Conclusion

The two approaches that we have described above, FO electrode recordings and development of scalp EEG-based detectors, provide opportunities to both directly record and indirectly identify silent mTL epileptiform abnormalities in AD. Both approaches have the potential to provide important windows into understanding the role of silent mTL epileptiform activity in AD, but will need to be further explored and validated for use in the AD population. While additional FO recordings will be essential for defining the types and frequency of silent mTL epileptiform abnormalities that can occur in AD patients, these recordings can also serve an important role in the development and validation of non-invasive, scalp EEG-based detectors of silent mTL epileptiform activity for use in AD patients. In the long run, scalp EEG-based detectors of silent mTL epileptiform activity have the potential to elucidate the prevalence, natural history, and role of silent mTL epileptiform activity in AD. Moreover, they could be instrumental for the development of new therapeutic approaches for AD, based on the modulation of silent mTL epileptiform activity.

## Author Contributions

AL, AC, and SC contributed to the intellectual development of the concepts and methods described. AL wrote the first draft of the manuscript. AL, AC, and SC contributed to manuscript revision, read, and approved the submitted version.

### Conflict of Interest Statement

The authors declare that the research was conducted in the absence of any commercial or financial relationships that could be construed as a potential conflict of interest.
